# In situ Precursor-Template Route to Semi-Ordered NaNbO_3_ Nanobelt Arrays

**DOI:** 10.1007/s11671-010-9757-0

**Published:** 2010-08-26

**Authors:** Junshu Wu, Dongfeng Xue

**Affiliations:** 1 State Key Lab of Fine Chemicals, Department of Materials Science and Chemical Engineering, School of Chemical Engineering, Dalian University of Technology, Dalian 116012, China

**Keywords:** Precursor template, NaNbO_3_ nanobelt, Semi-ordered array, Hydrothermal synthesis

## Abstract

We exploited a precursor-template route to chemically synthesize NaNbO_3_ nanobelt arrays. Na_7_(H_3_O)Nb_6_O_19_·14H_2_O nanobelt precursor was firstly prepared via a hydrothermal synthetic route using Nb foil. The aspect ratio of the precursor is controllable facilely depending on the concentration of NaOH aqueous solution. The precursor was calcined in air to yield single-crystalline monoclinic NaNbO_3_ nanobelt arrays. The proposed scheme for NaNbO_3_ nanobelt formation starting from Nb metal may be extended to the chemical fabrication of more niobate arrays.

## Introduction

One-dimensional (1D) nanostructures are receiving an ever-increasing amount of attention from researchers in various disciplines because of their unusual quantum properties to their bulk counterparts and potential use as building blocks for the next generation of nanoscale optical, electronic, photonic, and biological devices [1,2]. Ordered functional arrays or chemically defined surfaces with fascinating quantum behaviour are more attractive nanostructures owing to their applications in high-density memories, sensors, lasers, and photonic crystals. Although numerous efforts have been invested in developing simple and low-cost fabrication techniques for the growth of high-quality 1D materials in a relatively large scale [3-10], the ability to fabricate ordered 1D micro- and nanostructures in a desired pattern with controllable size and shape uniformity is a key challenge in enabling their improved technological applications and has opened up the minds of new generation of materials scientists about the potential of nanoscience and technology [11,12].

Alkaline niobates is one class of widely investigated ternary materials because of their optical, ferroelectric, and piezoelectric properties [13-19]. To date, many niobium-containing perovskite materials have been synthesized through various kinds of methods and exhibit wide applications in nonlinear optics, pyroelectric detectors, and optical memories, etc. NaNbO_3_ belongs to a technologically important group of perovskite materials, which comprises a three-dimensional framework of corner-sharing NbO_6_ octahedra with Na cations occupying their cavities [20-22]. Tilting of NbO_6_ octahedra at different temperature brings different phases (orthorhombic, monoclinic, and cubic phase). Generally, the shape of crystalline particles depends on their internal structures [23]. This means that materials with a cubic or pseudocubic structure will normally form isotropic particles in a thermodynamic decided process. Actually, till now there are few reports about high aspect ratio 1D NaNbO_3_ micro/nano structures, which provide a good system to study the size and dimensionality dependences of the physical properties. Herein, we exploit a facile precursor-template route to chemically fabricate NaNbO_3_ nanobelts. After a solid-phase transformation of Na_7_(H_3_O)Nb_6_O_19_·14H_2_O precursors in air, semi-ordered NaNbO_3_ nanobelt arrays were yielded without morphology deformation. In this proposed scheme, the aspect ratio and uniformity of the precursor and NaNbO_3_ nanobelts are controllable. Low concentration of NaOH in this process also avoids strong corrosive effect.

## Experimental

A typical synthesis was performed as follows. A piece of Nb foil (6 × 6 × 0.5 mm) was pretreated by sonication in ethanol for 10 min and laid flat in a Teflon-lined stainless steel autoclave (capacity, 30 mL). Twenty millilitres 1.0 M NaOH solution mixed with 3 mL H_2_O_2_ (PH = 13.2) was then filled into the autoclave that was sealed and put into an electric oven. The concentration of NaOH was changed from 0.5 to 1.5 M to tune the aspect ratio of precursor nanobelts. The temperature of the electric oven was set at 150–220°C and kept the reaction lasted for 10–24 h under autogenous pressure. After the autoclave was air-cooled to room temperature, Nb foil was dissolved completely, and the white sheet-like powders were collected, rinsed with deionized water, and dried at 40–60°C for 2–5 h in air. NaNbO_3_ nanobelt arrays could be obtained by calcining the precursor at 500–550°C for 1–4 h.

The as-prepared samples were characterized by an X-ray diffractometer (XRD) on a Rigaku-DMax 2400 diffractometer equipped with the graphite monochromatized Cu Kα radiation flux at a scanning rate of 0.02°/s in the 2θ range 5–80°. Scanning electron microscopy (SEM) images were taken with a JEOL-5600LV scanning electron microscopy, using an accelerating voltage of 20 kV. Energy-dispersive X-ray (EDX) microanalysis of the samples was performed during SEM measurements. The structures were investigated by transmission electron microscopy (TEM, Philips, Tecnai G220, operated at 200 kV). Thermogravimetric analysis and differential scanning calorimetry (TG/DSC, SDT Q600, TA) were employed to analyse the thermal behaviours of the synthesized precursor in N_2_ atmosphere at a heating rate of 10°C/min. UV–visible (UV–Vis) spectra of the samples were measured on a UV–Vis-NIR spectrophotometer (JASCO-V570). The photoluminescence (PL) spectra were measured at room temperature in the range of 310–700 nm using a Xe lamp with a wavelength of 290 nm as the excitation source. The infrared (IR) spectrum was measured by KBr pellet method (using a Nicolet NEXUS infrared spectroscopy) in the range of 400–4,000 cm^-1^.

## Results and Discussion

**Figure 1:**
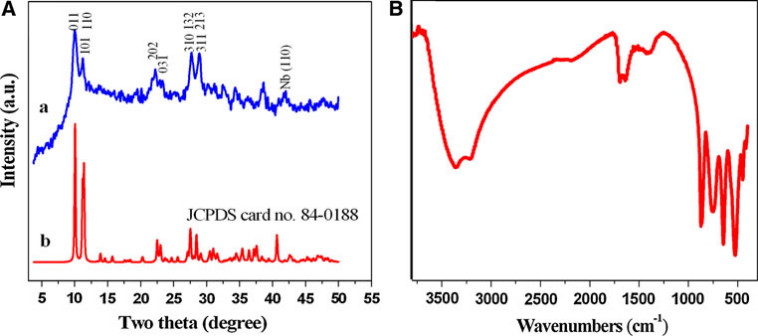
**a XRD patterns of Na_7_(H_3_O)Nb_6_O_19_·14H_2_O nanobelts**. The peak corresponding to remnant Nb is marked. **b** IR spectrum of Na_7_(H_3_O)Nb_6_O_19_·14H_2_O precursor.

Hydrothermal technique has been most popular and widely used in the synthesis of advanced materials of different disciplines owing to its advantages in terms of high reactivity of reactants, formation of metastable and low energy consumption. In our scheme, Na_7_(H_3_O)Nb_6_O_19_·14H_2_O nanobelts were firstly synthesized under mild hydrothermal conditions. XRD pattern of the obtained product is shown in Figure 1a. The major diffraction peaks can be indexed as the Na_7_(H_3_O)Nb_6_O_19_·14H_2_O with an orthorhombic lattice (JCPDS card no. 84-0188). The broad diffractive peaks are attributed to the nanosize of the sample. Moreover, a characteristic diffraction peak from remnant Nb foil is detected. The molecular structure of Na_7_(H_3_O)Nb_6_O_19_·14H_2_O is further supported by the solid-state IR spectrum (Figure 1b), which is in agreement with the literature values [24].

It has been reported that as the concentration of AOH (A = Na, K) increases in solution, the Lindquist ${\text{A}}_{8}^{+}{\left[{\text{Nb}}_{6}{\text{O}}_{19}\right]}^{8-}$ compound is salted out as a major product [25]. In each Lindquist ion of Na_7_(H_3_O)Nb_6_O_19_·14H_2_O, six NbO_6_ octahedra compose a bigger octahedron by sharing edges, Na atoms are scattered within the vacancies between the Lindquist octahedral sites. Further examination of the crystal structure of Na_7_(H_3_O)Nb_6_O_19_·14H_2_O reveals that the preferential orientation of the Na_7_(H_3_O)Nb_6_O_19_·14H_2_O is along *c*-axis. Considering that no templates and surfactants are used, it is the internal crystal structure that induces 1D growth of the Lindquist composite. Because of the coexistence of H_2_O_2_ and NaOH, alkaline oxidant solution is formed in the autoclave. The metal substrate is oxidated continuously by H_2_O_2_ into Nb_2_O_5_ which is subsequently dissolved by NaOH. Metastable phases often crystallize first at low temperature because their nucleolus may require lower free energy and lower supersaturation to form in the nucleation-controlled regime. Therefore, Na_7_(H_3_O)Nb_6_O_19_·14H_2_O is crystallized and precipitated out of the solution.

**Figure 2:**
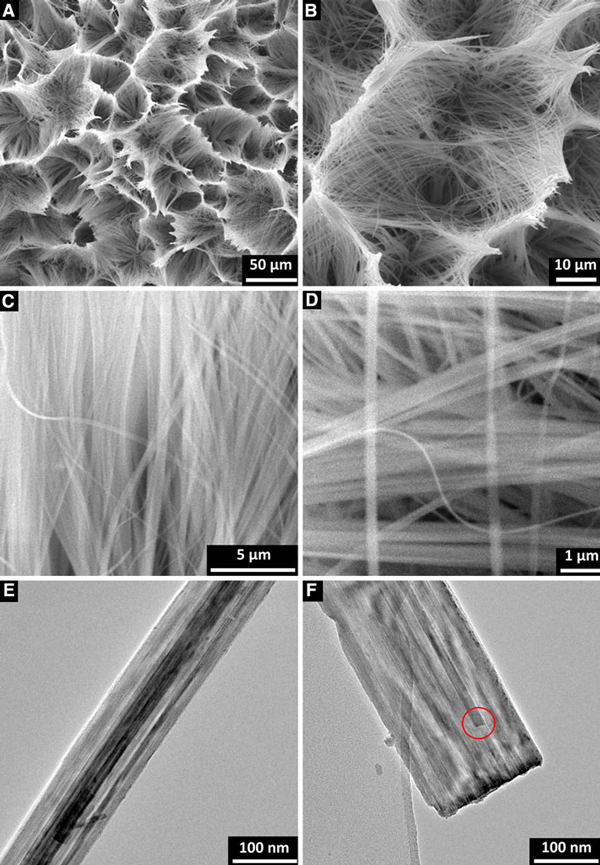
**a, b Low-magnification SEM images of Na_7_(H_3_O)Nb_6_O_19_·14H_2_O nanobelts, indicating the honeycomb-like micropattern**. **c**, **d** High-magnification SEM images. **e**, **f** TEM images of a single nanobelt, exhibiting contrasty stripes along the growth direction. The nanobelts are composed of many smaller size nanobelts shown with a *red circle*.

**Figure 3:**
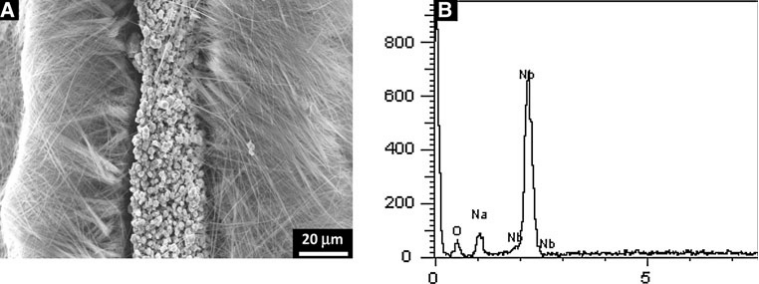
**a Side view of Na_7_(H_3_O)Nb_6_O_19_·14H_2_O nanobelt array, exhibiting that the nanobelts are typically ~50 μm in length**. **b** EDX spectrum of the irregular-shaped microcrystal layer where NaNbO_3_ nanobelts grow, indicating the presence of Na, Nb and O.

The low-magnification SEM images of Na_7_(H_3_O)Nb_6_O_19_·14H_2_O (Figure 2a, b) have been taken from randomly selected areas, and as such, these are representative of the overall sizes and shapes in the samples. It is seen that ultralong Na_7_(H_3_O)Nb_6_O_19_·14H_2_O nanobelt arrays are with honeycomb-like micropatterns. The length is in micrometer range. Furthermore, the high-magnification SEM images in Figure 2c, d reveal that these nanobelts are formed uniformly and compactly with typical widths of ~300 nm and thicknesses of ~80 nm. TEM and HRTEM images provide further insight into the microstructural details of belt-like nanostructures. Figure 2e shows that the nanobelt has a uniform width, and there are some contrasty stripes along the growth direction. A magnified image of a single nanobelt in Figure 2f exhibits clearly that the 1D structure is the belt-like aggregate morphology. The nanobelt bundles are essentially aligned in the same orientation and have different packing density resulting in the contrasty stripes in TEM observation. A cross-section of Na_7_(H_3_O)Nb_6_O_19_·14H_2_O nanobelt array is shown in Figure 3a. It is found that the nanobelts are typically ~50 μm in length, and they grow on an irregular-shaped microcrystal layer. EDX spectrum of the substrate in Figure 3b indicates the presence of Na, Nb, and O. Therefore, since the Nb metal foil is dissolved completely, it is a kind of sodium niobate substrate that induces the growth of Na_7_(H_3_O)Nb_6_O_19_·14H_2_O nanobelt.

**Figure 4:**
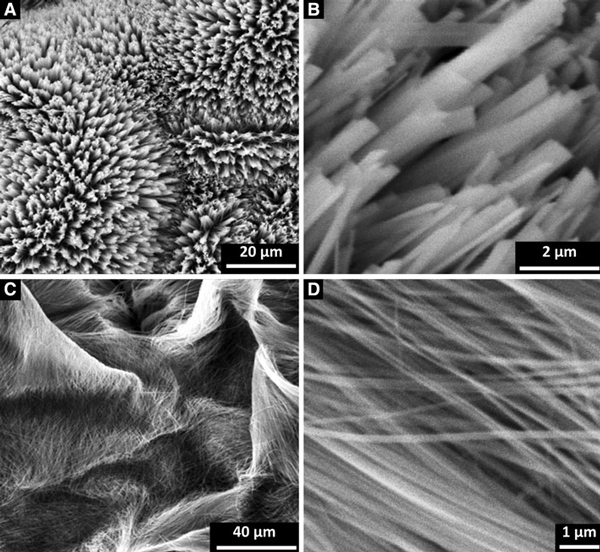
**SEM images of Na_7_(H_3_O)Nb_6_O_19_·14H_2_O precursor prepared at different NaOH concentrations: a, b 0.5–0.8 M, and c, d 1–1.5 M**.

We can control the aspect ratio and micropatterns of Na_7_(H_3_O)Nb_6_O_19_·14H_2_O structures by tuning NaOH concentration in the wet chemistry process. At a concentration of 0.5–0.8 M, hedgehog-like patterns are formed. The dense Na_7_(H_3_O)Nb_6_O_19_·14H_2_O microbars are with widths mostly less than 1 μm and thicknesses around 150 nm (Figure 4a, b). However, when the concentration is increased to 1–1.5 M, the nanobelts become so long that they gather compactly and overspread the substrate (Figure 4c, d). Therefore, we conclude that the alkaline concentration has a significant influence on morphology.

**Figure 5:**
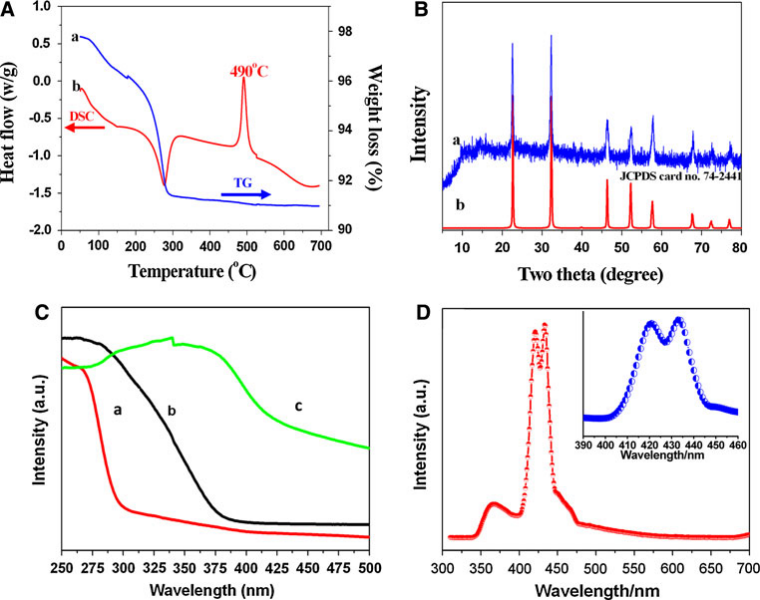
**a Weight change and heat flow recorded for Na_7_(H_3_O)Nb_6_O_19_·14H_2_O nanobelts**. **b** XRD pattern of NaNbO_3_ nanobelts (*a*) and the standard pattern of bulk NaNbO_3_ (*b*). **c** UV–Vis spectra of (*a*) Na_7_(H_3_O)Nb_6_O_19_·14H_2_O nanobelts, (*b*) NaNbO_3_ nanobelts, and (*c*) bulk NaNbO_3_. **d** Room temperature PL spectra of NaNbO_3_ nanobelts, the *inset* shows an enlarged spectrum.

The thermal decomposition process of Na_7_(H_3_O)Nb_6_O_19_·14H_2_O is shown in Figure 5a. As seen in TG curve, there is only one evident step involving dehydration. The weight of the sample significantly decreases in the temperature range of 80~290°C. Furthermore, between 290 and 515°C, the mass loss becomes quite slow and ceases at higher temperature (around 515°C). The total mass loss is about 9%, slightly smaller compared with standard value (11.3%). It is possible that some impurities, such as the microparticles below the nanobelt arrays, bring the difference. DSC plot for the decomposition recorded in nitrogen gas shows two peaks: one is endothermic event corresponding to the rapid release of H_2_O, and the other is exothermic peak at around 490°C corresponding to the transformation into NaNbO_3_ phase, which can be completed at around 515°C. Thermal decomposition of the precursor nanobelts under normal atmospheric conditions gives rise to the formation of a pure monoclinic NaNbO_3_ phase. As indicated in the Figure 5b, all the peaks in XRD pattern can be indexed well as the pure phase (JCPDS card no. 74-2441). During the thermal conversion process, NbO_6_ octahedra change from edge-sharing to corner-sharing. This structural difference has an important effect on the gap between the valence band and conductive band of niobates, which can be reflected in the optical absorption spectra of the as-prepared samples. UV–Vis spectra of the precursor, final product, and bulk NaNbO_3_ are shown in Figure 5c. The precursor has an absorption peak at around 250 nm (a) due to the Lindquist units including six edge-sharing NbO_6_ octahedra. However, the peak shifts to above 300 nm in final NaNbO_3_ product (b) that comprises corner-sharing NbO_6_ octahedra. The change may originate from the difference in Nb–O bond distances in configuration of NbO_6_ octahedra, which also further confirms that corner-sharing NbO_6_ octahedra are more stable than edge-sharing ones. When compared with UV absorption peak of bulk NaNbO_3_ (c) at around 362 nm, the optical absorption edge of NaNbO_3_ nanobelts shifts towards the lower wavelength, indicating an increase in band gap. Due to the quantum size effect in nanosized semiconductors, the band gap increases when the size of belt-like nanomaterials is decreased, resulting in a blueshift of absorption bands. PL spectra of NaNbO_3_ nanobelts were also been measured, as shown in Figure 5d. The spectra consist of a UV emission peak and two violet emission peaks in visible region. It is found that the UV peak position is at approximately 368 nm which can be attributed to free exciton emission. Two strong visible peaks dominate the PL spectra, which locate at λ = 421 and 433 nm. The spectra suggest that niobate framework is directly involved in the photoluminescence effect. The heat treatment increases the stability of Nb–O–Nb bonds and introduces different kinds of defect centres acting as traps for charge carriers, therefore increasing the probability for electrons to reach an electron trap, such as oxygen vacancy, and leading to the luminescence effect. The optical properties of NaNbO_3_ thus open up opportunities for exploiting advanced NaNbO_3_-based optical nanodevices.

**Figure 6:**
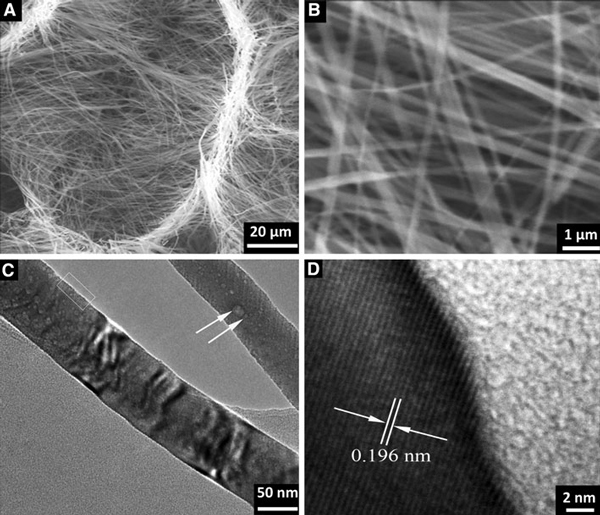
**a, b SEM images of NaNbO_3_ nanobelt at different magnifications**. **c** TEM image of a single nanobelt. The *arrow* in *top right* shows a surface pit. **d** HRTEM image of the selected area in **c**.

Evidence that the nanobelts have retained their morphology is shown in Figure 6. The NaNbO_3_ nanobelt arrays still have ordered honeycomb-like micropatterns (Figure 6a). A high-magnification SEM image (Figure 6b) indicates NaNbO_3_ nanobelt has the width of 0.1–0.5 μm. The surface is clean and without any sheathed amorphous phase. A ripple-like contrast is observed due to the strain resulting from the bending of the belt. In addition, there are some pits on the surface, which may be generated by the high-temperature heat treatment (Figure 6c). HRTEM image taken from the edge area of a NaNbO_3_ nanobelt reveals that it is structurally uniform single-crystalline phase without any obvious defects and dislocations (Figure 6d). The 2D lattice fringes are oriented approximately 45^°^ from the growth direction. The lattice-resolved image shows the fringes are separated by a distance of about 0.196 nm, which perfectly matches the lattice spacing of the (002) planes (1.959 Å) in the monoclinic NaNbO_3_ phase.

**Figure 7:**
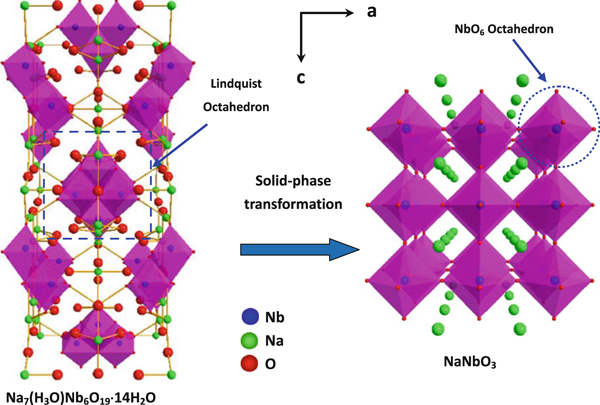
**Structural transformation from Lindquist precursor to NaNbO_3_**.

Those Lindquist ions in Na_7_(H_3_O)Nb_6_O_19_·14H_2_O extend along the [001] direction and build the backbone of the 1D structure. In subsequent calcination stage, the Lindquist units with the edge-sharing Nb–O polyhedra are ruptured to form more stable corner-sharing polyhedron groups. As the transitions mainly involve the breakage of chemical bonds such as Nb–O and rotation of NbO_6_ octahedra, the temperature as high as 500–550°C is needed to drive rate-limiting diffusion in solid-state phase conversion. It is well known that during the thermal dehydroxylation process, the water molecules are formed and lost between the two adjacent layers of hydroxyl ions. Under the high temperature, the dehydration process occurs quickly (as evidenced by the TG curve in Figure 5a) producing a great many atomic vacancies, which results in low thermal stability of Na_7_(H_3_O)Nb_6_O_19_·14H_2_O in the state. Therefore, to minimize the overall system energy and stability the crystal structure, the diffusion of Nb, O, and Na atoms is accelerated. During the structural transformation process, owing to the conventional six-coordinate microstructure, niobium atoms work as central atoms and coordinate with oxygen in the 1D precursor, then many small networks comprising corner-sharing NbO_6_^7-^ units generate as the crystalline nuclei. With the heat treatment process proceeds, a whole rearrangement atomic network is built on pre-existing nuclei in the restricted space of the precursor. That is, a steadier framework of corner-sharing NbO_6_ octahedra with Na atoms occupying the cavities is generated across the whole volume (Figure 7). This dehydration process requires long-range diffusion of Nb and Na atoms, and this reaction cannot be topochemical. However, in the heat treatment condition, sudden collapse of the precursor nanobelt can be avoided. As a result, the formation of NaNbO_3_ nanobelt derived from the atomic rearrangement in the crystal structure of Na_7_(H_3_O)Nb_6_O_19_·14H_2_O is observed during the decomposition process. It is noteworthy to point out, because of the nanosized diffusion distances for atoms moving between the contact areas, that the wire-like aggregates of Na_7_(H_3_O)Nb_6_O_19_·14H_2_O nanobelts can be converted into single-crystalline NaNbO_3_ nanobelts conveniently under high temperature. Moreover, the high temperature also brings lots of thermal defects in original sublattices, some of which may expand to the surface finally and result in some pits (see the area indicating by arrowhead in Figure 6c).

**Figure 8:**
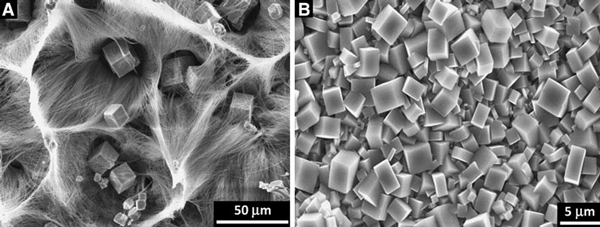
**a SEM image of the product prepared at 150–180°C for 20–24 h, indicating the coexistence of Na_7_(H_3_O)Nb_6_O_19_·14H_2_O nanobelts and NaNbO_3_ cubes**. **b** NaNbO_3_ nanocube film obtained at 200–220°C for 18–24 h.

All the above results reveal that the 1D characteristic of the precursor and the solid-state phase transformation process are all the important factors on the formation of perovskite NaNbO_3_ nanobelts. The existing 1D nanostructure serves as structural template from which NaNbO_3_ nanobelt can be readily generated. The size of the precursor is a critical determinant factor in governing the resultant shape of the final NaNbO_3_ product. Above a critical size, propagation of the reaction front is observed, and the basic morphology of the precursor is maintained [26]. During transformation process of the Na_7_(H_3_O)Nb_6_O_19_·14H_2_O nanobelt, the width of the reaction zone is not comparable to the size of this precursor, propagation of the reaction front can span, and the 1D nonequilibrium shape can be maintained. A second vital factor is post-temperature-induced phase transformation that drives oriented rearrangement of NaNbO_3_ nanoparticles into single-crystalline nanobelts. The precursor undergoes solid-phase reactions rather than continues to grow under hydrothermal circumstance, no dissolution and atom-by-atom recrystallization process happens, which prevents potential shape evolution of the 1D precursor. Therefore, perovskite formation in solution phase can be avoided using short treat time. Increase in reaction time results in lots of NaNbO_3_ cubes, as shown in Figure 8a. The controllable surface structures are further shown in Figure 8b. When increasing reaction temperature from 150–180 to 200–220°C, stable NaNbO_3_ perovskite is formed, and no 1D nanostructure is obtained. The higher temperature affords adequate energy to overcome the activation energy and the reaction barrier in the formation of perovskite structure. This also affords a facile way to change the surface structure of niobate films.

## Conclusions

We have successfully developed a facile precursor-template route to chemically fabricate dense semi-ordered NaNbO_3_ nanobelt arrays with tunable aspect ratio, which may be thermodynamically inaccessible structural and morphological features. During the thermal conversion process, atoms gradually rearrange in the restricted space of 1D Na_7_(H_3_O)Nb_6_O_19_ 14H_2_O precursor until single-crystalline NaNbO_3_ nanobelt forms without loss of the original shape. The study facilitates to advance the understanding of the crystal phase control and transformation during solid-state reactions. We also established the controlled organization of the film surface with NaNbO_3_ nanocubes, which may be also useful for optical and piezoelectric devices. The proposed chemical strategy for NaNbO_3_ film formation may be extended to the fabrication of more niobate arrays.
